# Bubbles Moving in Blood Flow in a Microchannel Network: The Effect on the Local Hematocrit

**DOI:** 10.3390/mi11040344

**Published:** 2020-03-26

**Authors:** David Bento, Sara Lopes, Inês Maia, Rui Lima, João M. Miranda

**Affiliations:** 1 CEFT, Faculdade de Engenharia da Universidade do Porto (FEUP) Rua Dr. Roberto Frias, 4200-465 Porto, Portugal; davidbento@ipb.pt (D.B.); rl@dem.uminho.pt (R.L.); 2 Polytechnic Institute of Bragança, ESTiG/IPB, C. Sta. Apolónia, 5300-857 Bragança, Portugal; sara_lopeslh@hotmail.com; 3 Instituto Superior Técnico, Universidade de Lisboa, Av. Rovisco Pais, 1049-001 Lisboa, Portugal; inesmaiag@tecnico.ulisboa.pt; 4 MEtRICS, Mechanical Eng. Dep., University of Minho, Campus de Azurém, 4800-058 Guimarães, Portugal

**Keywords:** blood flow, gas embolism, microcirculation, bifurcations, network

## Abstract

Air inside of blood vessels is a phenomenon known as gas embolism. During the past years, studies have been performed to assess the influence of air bubbles in microcirculation. In this study, we investigated the flow of bubbles in a microchannel network with several bifurcations, mimicking part of a capillary system. Thus, two working fluids were used, composed by sheep red blood cells (RBCs) suspended in a Dextran 40 solution with different hematocrits (5% and 10%). The experiments were carried out in a polydimethylsiloxane (PDMS) microchannel network fabricated by a soft lithography. A high-speed video microscopy system was used to obtain the results for a blood flow rate of 10 µL/min. This system enables the visualization of bubble formation and flow along the network. The results showed that the passage of air bubbles strongly influences the cell’s local concentration, since a higher concentration of cells was observed upstream of the bubble, whereas a lower local hematocrit was visualized at the region downstream of the bubble. In bifurcations, bubbles may split asymmetrically, leading to an uneven distribution of RBCs between the outflow branches.

## 1. Introduction

Gas bubbles flowing in the bloodstream can be a dangerous and uncontrolled occurrence or a useful and controlled therapeutic procedure. Gas bubbles can be formed when a patient is subjected to abrupt pressure changes (e.g., scuba diving [1], space operations [2], and high altitude flight [3] incidents), or due to pathologic conditions (e.g., heart valves damage [4,5]). On the other hand, air can be injected, accidentally or deliberately, in a patient during medical procedures (e.g., surgeries [6,7], hemodialysis [8], and embolotherapy [9]). If uncontrolled, the bubbles can limit the oxygen supply, which can lead to tissue damage, disability, or even death [10]. Nevertheless, bubbles can also be used for medical diagnosis and treatments. One example is the use of bubbles as probes for image contrast enhancement at clinically relevant ultrasound frequencies [11]. Another therapeutic strategy is the occlusion of vessels in tumorous tissues, limiting the blood supply and inducing tumor necrosis [12]. Microbubbles also have the potential to be used as drug carriers [13]. Consequently, it is important to study in detail the mechanisms involved on the formation of bubbles, as well as bubbles behavior on the blood vessels and how they influence the blood flow [14]. It is known that the size, composition, and shape of the bubbles will influence their behavior [14] since they flow as tubular bubbles in small vessels [9,15]. Larger bubbles tend to be entrapped in microcirculation [12], while smaller bubbles can be dissolved in blood or be lodged on bifurcations or in small branches [9]. The lodging and adhesion of bubbles is also greater on regions with blood flow disturbances such as bifurcations [16,17].

In microchannels and microvessels, the blood stream tends to form cell free layers (CFL) [18,19,20]. The CFL plays an important role in balancing nitric oxide (NO) production by the endothelium and NO scavenging by red blood cells (RBCs) [21]. The RBC’s concentration, i.e., the hematocrit (Htc), can influence the CFL and the formation of RBC aggregates. An increase in the Htc leads to a reduction of the CFL [18] and high Htc and low flow rates lead to larger aggregates [22]. On the other hand, microbubbles promote the formation of a CFL around it [23]. Consequently, it is possible that bubbles present in microcirculation have an influence on physiological process such as oxygen supply to the tissues and NO scavenging by RBCs [21].

Several pathological conditions, including arterial hypertension, ischemia, inflammation, and diabetes, can be detected and monitored by analyzing the blood and blood cells of the patient [24]. Hence, microfluidic devices are important assets for those analyses due to the precise spatio-temporal control and their small dimensions [25]. Those devices depend on the precise control and manipulation of the working fluid that is being tested. However, a major problem of these devices is the high tendency for clogging, jamming, and possible blockage due to bubbles and cell entrapment [26,27].

When flowing in microchannels, the bubbles promote long vortices [28,29] downstream and upstream. These vortices can be observed when the streamlines are represented in the bubble reference frame. In vitro studies have shown that the vortices lead to intricate variations, both spatial and temporal, of the distribution of the RBCs throughout the vessels, affecting the local rheology and transport processes [21]. In another study, it was shown that microbubbles induce non-uniform cell concentration in biomedical microdevices [23]. In these previous works [21,23], the bubbles moved in co-current with the blood stream along simple straight microchannels. However, the geometries more commonly found in microcirculation are complex networks composed by several bifurcations, and the effect of bubbles in RBC distribution in networks is still lacking. These studies could contribute to understand how bubbles can block capillary networks or lead to a non-uniform distribution of cells, a process that may lead to ischemia.

In this work, a microchannel network comprising three branch levels is used to mimic a region of the capillary system. Microbubbles are injected in the in vitro blood stream flowing through the network. The flow of the bubbles in co-current with blood is visualized through a microscope and recorded through a high-speed camera. The effect of bubbles in the RBC distribution is analyzed at several positions of the microchannel network.

## 2. Materials and Methods 

### 2.1. Working Fluids and Network Geometry 

**Figure 1:**
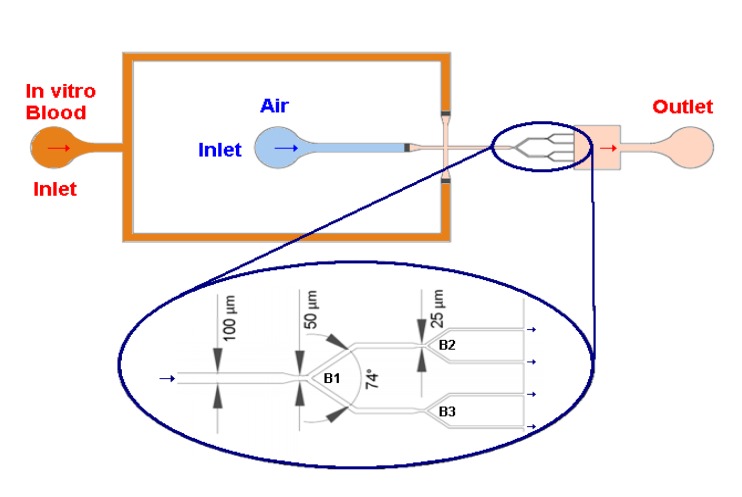
Schematic diagram of the microfluidic system and main dimensions of the network micro-channel used in this study: B1 is the first level bifurcation and B2 and B3 are second level bifurcations.

Two working fluids with Dextran 40 (Dx 40), containing about 5% and 10% of ovine RBCs, were prepared. The blood was collected from a healthy sheep using a tube containing ethylenediaminetetraacetic acid (EDTA) to prevent coagulation (According to the ethics committee of "Unidade Local de Saude do Nordeste", (ULSNE 2014-02-07), all experiments were conducted after ensuring that the in vitro blood experimental protocols were appropriate and in compliance with the EU directives 2004/23/CE, 2006/17/CE and 2006/86/CE.). Compared to humans’ RBCs, sheeps’ RBCs have a similar biconcave shape but they are smaller with an average major diameter of about 5 µm [30]. The RBCs were separated from the blood by centrifugation and washed twice with a physiological saline solution. More details can be found in previous works [13,15]. The geometry used in this work (Figure 1) comprises an inlet for air and another for the working fluid, a flow focusing region for bubble formation, a network of successive bifurcations, and an outlet. The depth of the microchannels is 50 µm. This geometry was used to manufacture the microfluidic device in PDMS through conventional soft-lithography [31] from SU-8 molds [32,33,34] (Microliquid, Mondragon Spain).

Several studies [35,36] indicate that Dextran influences or induces the aggregation of RBCs and, consequently, influences the blood rheological properties. However, the Dextran used in those works was a high molecular weight polymer, such as Dextran 500 [35] or Dextran 2000 [36]. In our work, we used a Dextran 40, a lower molecular weight polymer that avoids not only the aggregation and jamming of the RBCs within the microchannels but also their sedimentation [37,38].

### 2.2. Experimental Set-Up

**Figure 2:**
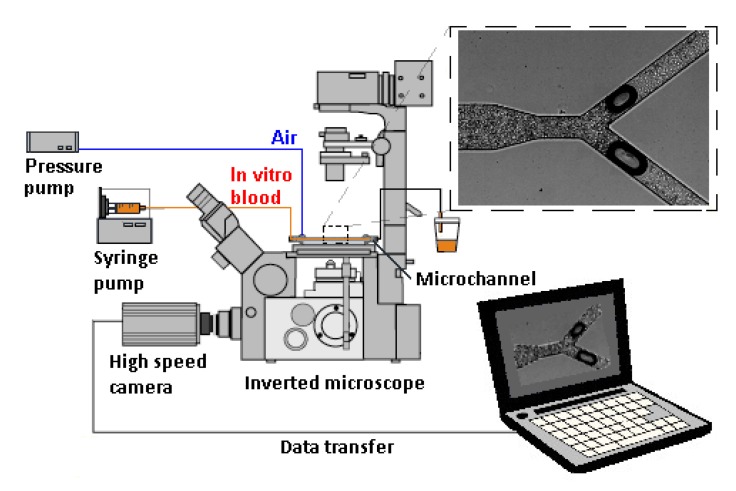
Schematic diagram of the experimental set-up used in this study.

The experimental set up comprises an inverted microscope (IX71, Olympus, Tokyo, Japan) combined with a high-speed camera (Fastcam SA3, Photron, San Diego, CA, USA) (see Figure 2). The PDMS microchannel was placed on the stage of the inverted microscope, and a syringe pump (PHD ULTRA, Harvard Apparatus, Holliston, MA, USA) was used to control the flow rate of the in vitro blood. In this study, the flow rate was set to 10 µL/min. Additionally, the air pressure was controlled by an air pump (Eleveflow PG1113, Fluigent, Paris, France). By using this latter pump, air droplets were injected into the microfluidic device in a well-controlled way. The air pump imposed a pressure of 210 mbar, the minimum pressure required to inject a bubble in the microfluidic device for the flow rate selected.

In order to characterize the flow conditions in the network, the following variables were estimated: average fluid velocity and wall shear rate. The average fluid velocity, *v*, is given by:

(1) $$v=\frac{Q}{A}$$

where *A* is the cross-section area and *Q* is the flow rate. The wall shear rate, *SR*, was estimated by:

(2) $$SR=\frac{v}{L}$$

where L is the minimum dimension between the walls of the microchannel. 

The fluid velocity and the wall shear rate are constant throughout the network and equal, respectively, to 0.033 m/s and 666.7 s^−1^. Hence, the flow conditions used in our network are similar to the conditions in microcirculation observed in vivo [39,40,41]. Typical average shear rate values observed in vivo in microcirculation can vary from 200 s^−1^ up to 2000 s^−1^. 

### 2.3. Image Processing

The images were obtained at the midplane of the microchannel with a frame rate of 2000 frames/second. After selecting a region of interest (ROI), the average tonality of the pixels in the ROI through time was determined using the Plot z-axis profile tool of the ImageJ software (V: 1.52a, National Institute of Health, Bethesda, MD, USA). The tonality plots (tonality versus time) were used to assess the effect of the air bubble on the local Hct.

## 3. Results and Discussion

**Figure 3:**
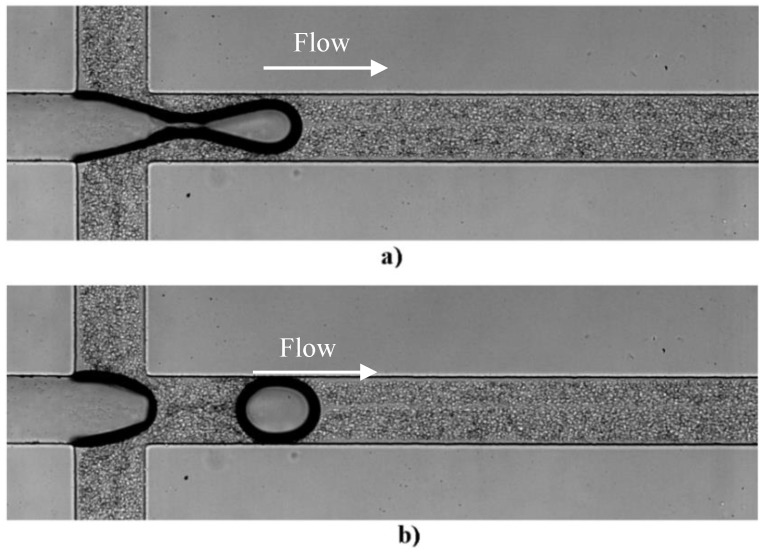
Sequential images of the formation of an air bubble: (**a**) bubble before detachment and (**b**) bubble after detachment (see Supplementary Video 1).

**Figure 4:**
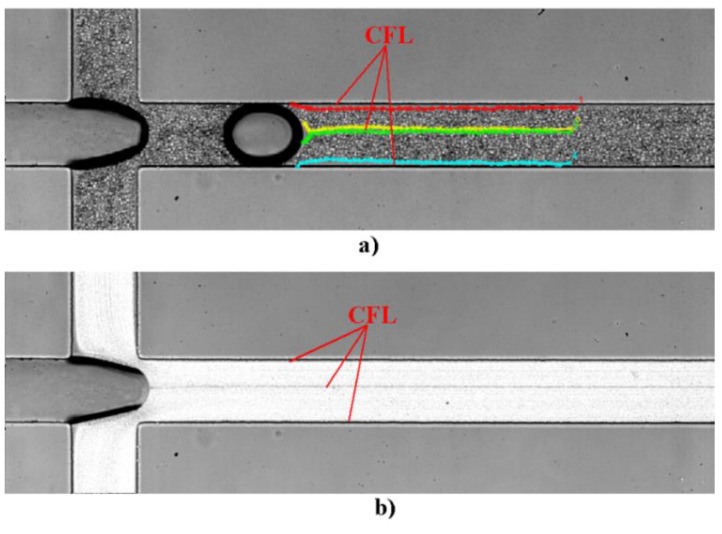
The different cell free layer (CFL) around the walls of the microchannel and in the middle of the channel by the processing image (**a**) MtrackJ and (**b**) Zproject by the ImageJ software.

The bubbles are formed at the flow focusing region located at the inlet of the first channel of the network. The formation of a bubble can be observed in Figure 3. Figure 3a shows the bubble before detachment whereas Figure 3b) shows the bubble after detachment. A CFL is clearly observed not only around the walls of the microchannel but also in middle of the channel due to the bubble formation, as can be seen in Figure 4. These results corroborate our previous findings performed in a simple geometry [13].

**Figure 5:**
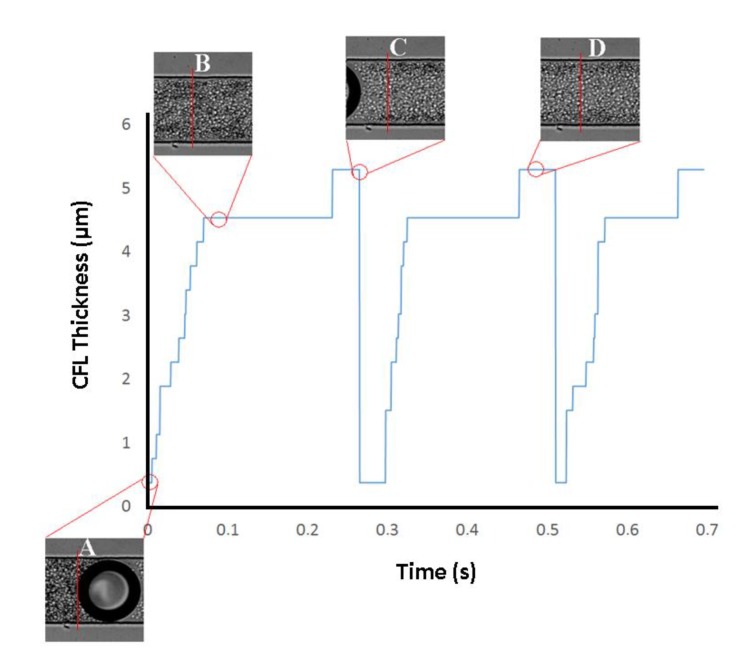
CFL thickness over time in the top wall of the channel.

One of the oldest and most interesting hemodynamic phenomenon was reported by Fahraeus and Lindqvist who observed that for narrow tubes (<300 μm) both hematocrit (Hct) and apparent viscosity of blood declines with decreasing diameter [42,43]. The physical reason behind this phenomenon is the tendency of RBCs to migrate toward the centre of the microtube, resulting in a marginal CFL at regions adjacent to the wall. In the present study, a CFL was clearly observed around the walls when there was no bubble within the microchannel. However, it was interesting to notice that this CFL disappears when the bubble passes through the flowing blood. Viewing Figure 5 allows for understanding of the Fahraeus and Lindqvist phenomenon, through the behavior of CFL thickness over time.

As is possible to see in Figure 5, due to the presence of the air bubble and the Hct behind the bubble, there is no CFL (point A in Figure 5) in the wall of the channel. When the bubble passes, there is a gradual formation of the CFL over time, until it stabilizes (point B in Figure 5). In addition, the approach of the air bubble causes a reduction in the Hct within the microchannel, which promotes a slight increase of the CFL thickness (point D in Figure 5).

**Figure 6:**
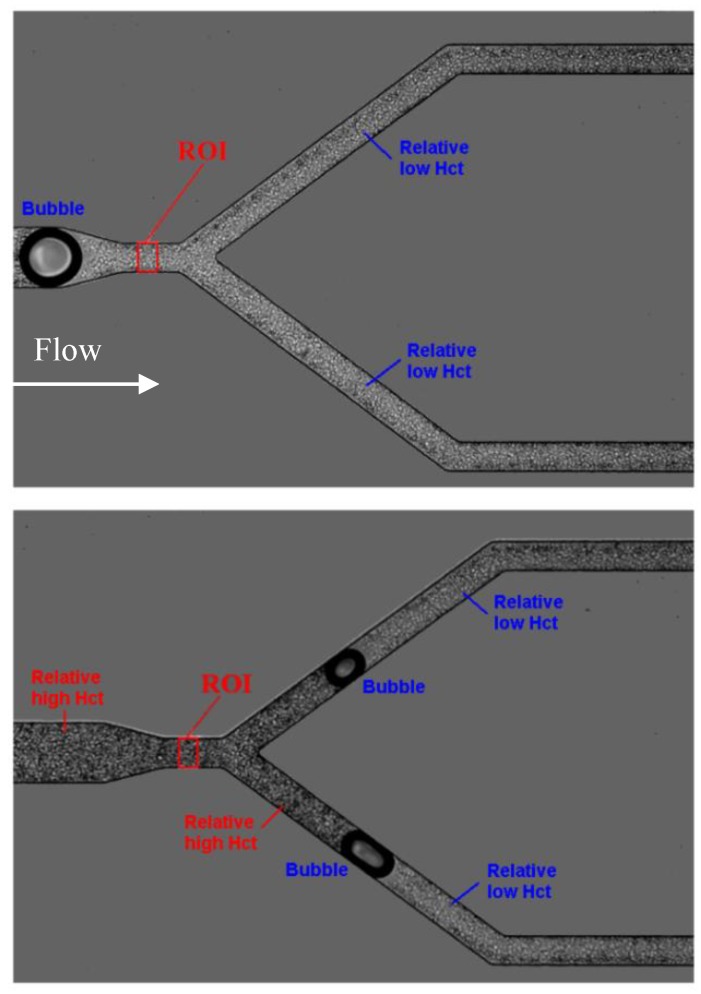
Influence of air bubbles on the local hematocrit (Hct) for a flow rate of 10 µL/min and 10% Hct at the first bifurcation (B1) (see Supplementary Video 2).

In the present study, we have performed flow measurements in more complex geometries having microchannel networks. In this way, the flow visualizations were performed along the microchannel network and all the videos were carefully examined. Overall, it was noted that air bubbles clearly influence the local Hct located downstream and upstream of them. Figure 6 shows this phenomenon at two different instants.

**Figure 7:**
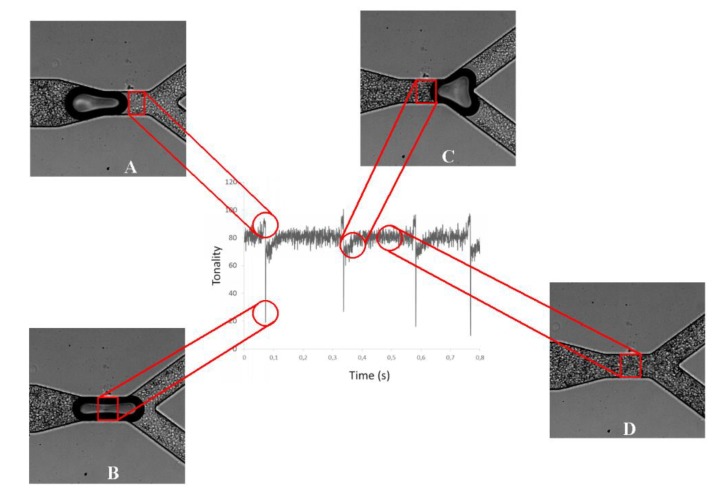
Application of the plot Z-axis profile function at the selected region of interest (ROI), for a Hct of 10% and at the first bifurcation (B1).

The tonality is not only related to Hct inlet concentration but also to bubble presence in the ROI. In general, for a Hct at an inlet of 10%, the average pixel intensity is constant when the bubble is far from the ROI, corresponding to the undisturbed average Hct. When the intensity reaches a peak, (point A of Figure 7) it means that a bubble is approaching, and it indicates a decrease of the average Hct at the ROI. When the air bubble passes through the ROI, the tonality decreases drastically (point B of Figure 7). After the bubble passage, the tonality tends to increase (point C of Figure 7) until it stabilizes and returns to the values initially reported. Thus, these results show that just after the air bubble passes through the ROI, there is an increase of the local Hct, which tends to decrease until it stabilizes and returns to its initial values. In addition, these results clearly show that the bubble affects the amount of cells flowing at its downstream region and, consequently, is likely to have a strong impact on blood mass transport mechanisms happening in microcirculation.

**Figure 8:**
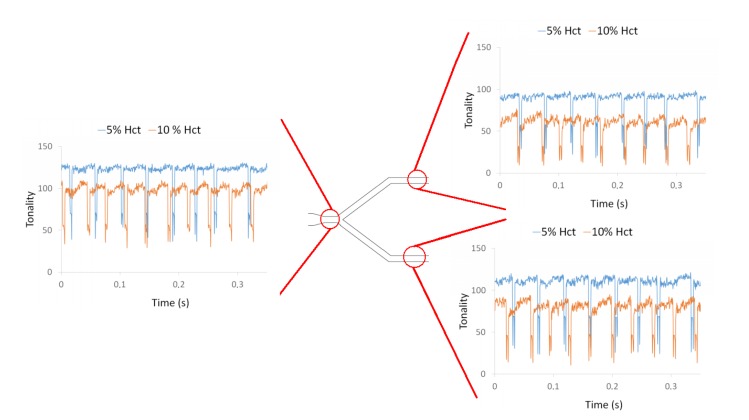
Influence of air bubbles on the local Hct for a flow rate of 10 µL/min for 5% Hct and 10% Hct at the first bifurcation (B1).

By applying the plot Z-axis profile function, the effect of the bubble was investigated at different locations of a microchannel network and for two different Hct, i.e., 5% and 10% Hct. From Figure 8 it is possible to observe the influence of the air bubbles on the local Hct for both RBC concentrations at the first bifurcation of the microchannel network. These measurements show clearly that the local Hct is higher upstream and lower downstream of the bubble. This difference was more evident for 10% Hct than for 5% Hct. Hence, the effect of the air bubbles on the blood flow behavior and local Hct tends to increase with the Hct. Additionally, these results show that the distribution of Hct downstream of the bifurcation is asymmetric. At the bottom channel of the bifurcation, the tonality is higher than that of the top channel, i.e., the local Hct in that bottom channel is lower than the Hct at the top channel. This unbalance in Hct is likely to be due to asymmetric bubble splitting at the apex of the bifurcation.

**Figure 9:**
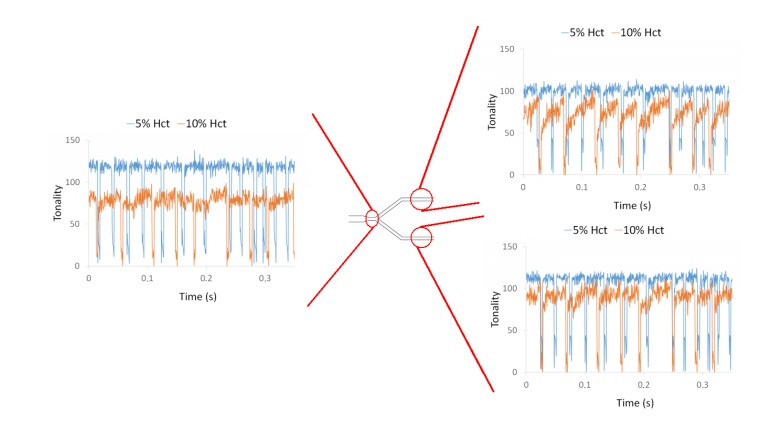
Influence of air bubbles on the local Hct for a flow rate of 10 µL/min for 5% Hct and 10% Hct in bifurcation B2.

Figure 9 shows the effect of the air bubbles on the local Hct at the bifurcation B2 of the microchannel network. The flow phenomena are similar to the previously observed first bifurcation (B1). The local Hct is affected by the air bubbles and the effect increases with the Hct. Once again, it was found an asymmetric distribution of the Hct due to the asymmetric bubble splitting at the bifurcation apex.

**Figure 10:**
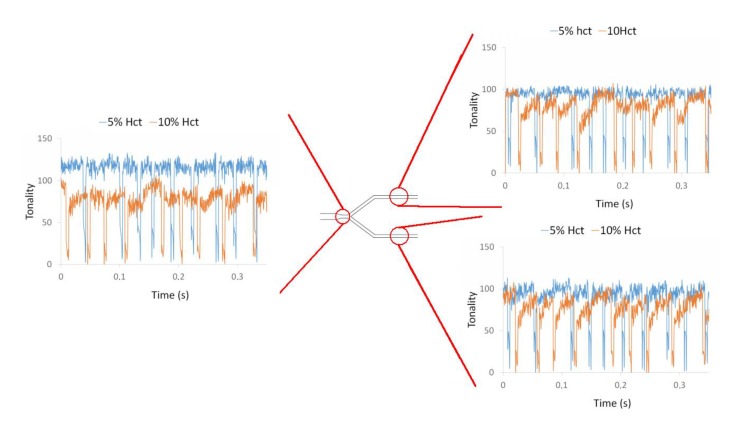
Influence of air bubbles on the local Hct for a flow rate of 10 µL/min for 5% Hct and 10% Hct at bifurcation B3.

Finally, Figure 10 shows the results obtained in the bifurcation B3. Again, at this bifurcation, the qualitative results and conclusions were similar to the ones obtained for the other bifurcations. However, at the outlet branches downstream to bifurcations B2 and B3, the tonality of the 10% Hct is higher than in the branches upstream to those bifurcations. The tonality is, in some parts of the outflow branches, almost coincident with the tonality obtained with 5% Hct. This phenomenon could be due to the reduction of the width of the microchannel, and consequently to the Faharaeus effect and Faharaeus–Lindqvist effect.

**Figure 11:**
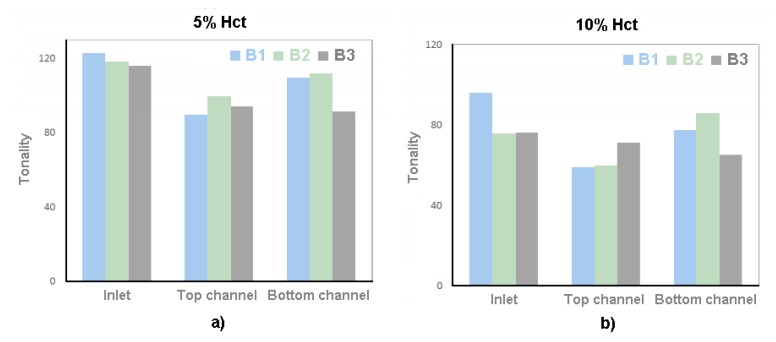
Tonality for (**a**) case 1, 5% Hct, and (**b**) case 2, 10% Hct.

**Figure 12:**
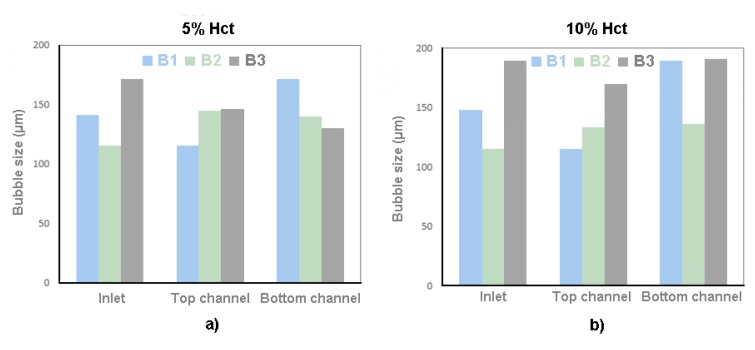
Bubble size for (**a**) case 1, 5% Hct, and (**b**) case 2, 10% Hct.

In order to understand whether the dimensions of the bubble would influence the local Hct, the dimensions of the bubble were measured and correlated with the intensity of pixels after the bubbles have passed through the ROI. Figure 11 shows the intensity of pixels after bubbles and Figure 12 is the results of the dimensions of the bubble.

Through the results present in Figure 11, it is possible to verify that case 1 presents a higher tonality than case 2. These results show that in case 1, with a feed Hct of 5%, there is a lower concentration of RBCs than in case 2, with a feed Hct of 10%.

The fact that, in all the bifurcations, the branches have a lower tonality than the inlet does not mean that there is an increase in the Hct in that region. In contrast, it means that, after air bubble collides with the bifurcation apex and divides, there is an accumulation of RBCs behind the bubble. Due to the fact that the division of the bubbles is asymmetrical, i.e., they do not present the same dimensions after the division; it also means that there is no symmetrical distribution of the RBCs by the branches of the bifurcation. This explains the difference of tonality between the branches.

Knowing that the B2 inlet is the top channel of B1 and the B3 inlet is the bottom channel of B1, as can be seen in Figure 1, it appears that there is an increase in the tonality in these channels. This happens because as the bubbles advance along the channel, the Hct existing behind them tend to stabilize.

Through the results present in Figure 12, it appears that there is no direct relationship between bubble size and the concentration of RBCs behind the air bubbles.

## 4. Limitations and Future Perspectives

In this study, it was shown that the bubble clearly disturbs the local Hct in the vicinities of the bubbles, and as a consequence the blood flow in microcirculation. However, in this study it was not possible to quantify in an accurate way the variations of the local Hct that was clearly observed from our experimental visualizations (see Supplementary Video 2). It is well known that by using a conventional microscopy system, the entire volume of the blood flow is illuminated, which leads to high measurement error due to the detection not only from the out of focus emitted light, but also the light from the focal plane of interest. Hence, in order to quantify the local Hct in a reliable way, it is recommended to use a confocal microscopy system [44,45,46]. Another possible alternative is by using an optical absorption spectrophotometric method. Recently, Faustino et al. [47] have performed a study with this technique and they were able to detect differences between blood samples with different concentrations. We believe that this optical method will be an excellent reliable approach to measure the local Hct in microfluidic devices.

In the experiments recorded throughout this work, the bubble breaks up at the apex of the bifurcation. In some of the break-up events, asymmetries are clearly observed. These asymmetries result from small differences in the downstream flow, due to geometrical imperfections or to the presence of bubbles in the downstream channels. It is known from literature [48] that when the geometrical or flow asymmetries are larger, bubble break-up is even more asymmetric, and in some cases, the bubble may flow unsplit to the channel with the higher flow rate. Future experiments in networks comprised of asymmetric bifurcations, with asymmetric flow distribution, will probably reveal a more complex scenario than the one observed in the present work, as a more pronounced bubble splitting will lead to a more pronounced asymmetric distribution of RBCs.

## 5. Conclusions

The objective of this work was to investigate the influence of air bubbles in the local Hct when flowing through a microchannel network comprised by successive bifurcations. The results obtained demonstrated that air bubbles flowing in the microchannel strongly affect the local Hct at the region ahead and behind them. When a bubble flows, the local Hct decreases at its downstream region and increases at its upstream region. After a certain time, the Hct tends to decrease until it reaches a steady Hct, i.e., without the effect of any bubble. These results indicate that bubbles flowing in microvessels or microchannels clearly affect the amount of cells flowing at its downstream region. Consequently, it is likely that they have a strong impact on the blood mass transport mechanisms happening in microcirculation and biomedical microdevices.

From this work it is also possible to conclude that the bifurcation, most of the time, produces air bubbles with different sizes and, as a consequence, tends to promote asymmetric Hcts along the subsequent branches of a microchannel network. Due to the effect of bubbles in the Hct, bubble splitting at bifurcations has direct implications on RBCs distribution between branches and may have important consequences to the development of ischemia due to gas embolisms.

## Supplementary Materials

The following are available online at https://www.mdpi.com/2072-666X/11/4/344/s1, Video S1: Sup_Video_1, Video S2: Sup_Video_2.
